# Apathy as a determinant of health behaviors in older adults: Implications for dementia risk reduction

**DOI:** 10.1002/dad2.12505

**Published:** 2023-11-23

**Authors:** Fleur Harrison, Moyra E. Mortby, Karen A. Mather, Perminder S. Sachdev, Henry Brodaty

**Affiliations:** ^1^ Centre for Healthy Brain Ageing (CHeBA) Discipline of Psychiatry & Mental Health Faculty of Medicine & Health, UNSW Sydney Sydney New South Wales Australia; ^2^ School of Psychology, UNSW Sydney Sydney New South Wales Australia; ^3^ Neuroscience Research Australia Sydney New South Wales Australia; ^4^ UNSW Ageing Futures Institute, UNSW Sydney Sydney New South Wales Australia; ^5^ NeuroPsychiatric Institute Prince of Wales Hospital Randwick New South Wales Australia; ^6^ Older People's Mental Health Service Prince of Wales Hospital Randwick New South Wales Australia

**Keywords:** affective determinants, amotivation, anergia, apathy, dementia risk reduction, depression, exercise, fatigue, health behaviors, lifestyle risk index, modifiable risk factors, negative symptoms, neuropsychiatric symptoms, physical activity

## Abstract

**INTRODUCTION:**

Long‐term improvements in physical inactivity and other behavioral risk factors are integral to dementia risk reduction; however, sustained behavior change is challenging. Apathy, depression, and fatigue may impact engagement in health behaviors, but their presentation overlaps. This study investigates whether these symptoms are differentially associated with multiple health behaviors.

**METHODS:**

In 1037 community‐dwelling older adults without dementia (aged 70–90, 55% women), regression analyses examined apathy, depression, and fatigue as predictors of health behaviors (physical activity, diet, alcohol, smoking) and a behavioral risk index.

**RESULTS:**

Apathy was associated with reduced physical activity and alcohol use, and one or multiple behavioral risk factors. No or inconsistent relations were found between depression or fatigue and health behaviors.

**DISCUSSION:**

Apathy is relevant to multiple health behaviors and should be considered when designing health promotion for older adults, including interventions for dementia risk reduction. Findings highlight the importance of distinguishing apathy from comorbid symptoms.

**Highlights:**

- Novel theory‐based perspective on behavioural risk factors for dementia.
- Higher apathy predicted less physical activity and alcohol use, and increased odds of lifestyle risk factors.
- Depressive symptoms were not associated with any health behavior.
- Apathy may be a determinant of multiple health behaviors in older adults, distinct from depression and fatigue.
- Considering apathy in precision prevention of dementia appears warranted.

## 1 INTRODUCTION

Sustained improvements in health behaviors are crucial to tackle global burden of diseases such as dementia, which represent decades of accumulated risks.^1^ Addressing modifiable behaviors including physical inactivity, smoking and alcohol use, along with other risk factors including obesity, hypertension, and diabetes, will prevent at least 40% of risk for dementia.^1^ However, a key challenge for dementia risk reduction is that health behaviors are notoriously difficult to change, and even more so to sustain.^2^ Standard behavior change interventions based on dominant theories of health behavior science have only modest, short‐term efficacy.^3^ Global burden of disease due to behavioral risk factors has not significantly improved over time, despite long‐term public health initiatives.^4^ Novel approaches are needed.

Affective determinants may play an important role in health behaviors.^5^ For example, stress and negative affect predict less physical activity the next day, while psychological well‐being predicts increased activity over a decade. Affective symptoms such as depression, fatigue, and apathy, which are highly prevalent in older people,^6^ may act as barriers to engaging in healthy lifestyles.^7^, ^8^ This has received little attention to date. As these symptoms overlap and are often subsumed in standard depression questionnaires,^9^ yet have important differences, it is critical to consider them separately.^10^ Apathy and depression differ in neurobiology, treatment, and prognosis, despite being commonly comorbid.^11^

Apathy, in particular, warrants consideration as a determinant of health behaviors. It is a symptom or syndrome defined as a reduction in motivation or goal‐directed behavior that occurs across the neurocognitive spectrum.^12^ In healthy older adults, prevalence estimates vary widely from 1.4% to 29.5%,^13^, ^14^ depending on age, the measurement tool used, and whether self‐ or informant perspectives are taken.^15^ Nonetheless, apathy demonstrably impacts multiple life domains, including quality of life, function, and frailty,^16^, ^17^ and is implicated in mortality, dementia, and cardiovascular disease outcomes, independent of depression.^18^, ^19^ That reduced motivation affects health behaviors has robust face validity: motivation is one of five themes underlying *all* theoretical explanations in the field,^2^ and was reported consistently by older adults as a key determinant of physical inactivity.^20^

Our aim was to investigate apathy as a determinant of health behaviors in older adults. Despite increasing evidence on neurobiological, vascular, and clinical correlates of apathy, there remains limited research on its behavioral correlates. No study to date has investigated apathy in relation to the “Big 4” health behaviors—physical activity, dietary intake, alcohol use, and smoking—which are major causes of non‐communicable diseases.^21^ Of these, physical activity has received most attention, with apathy consistently associated with reduced self‐report^22^, ^23^ and objective measures (motor inactivity, slower walking speed, restricted lifespace).^22^, ^24^ By contrast, evidence linking apathy with alcohol or smoking is scant and inconsistent,^25^, ^26^ and diet has been little investigated. Moreover, multiple health behaviors (physical inactivity and smoking) acted as mediators between apathy and incident cardiovascular disease.^23^ Taken together, findings to date indicate a potential impact of apathy on a range of health behaviors, which may act as a mechanism to poorer health outcomes.

Our study builds on this evidence, using cohort data of 1037 older adults without dementia. The objectives are to (1) investigate associations between two apathy measures (self‐ and informant‐report) and individual health behaviors (physical activity, diet, alcohol use, smoking), and (2) explore apathy in relation to multiple health behaviors, by quantifying its association with a behavioral index. Combining behaviors into a single risk index may better reflect relations between behaviors in real‐life contexts,^27^ and help address an important gap in knowledge on multiple health behaviors.^21^ We assess whether associations are independent of comorbid affective symptoms (depression, fatigue), and explore whether sex moderates associations, given likely sex differences in apathy and health behaviors. For this cross‐sectional research, we hypothesize that greater apathy will independently predict reduced levels of health behaviors (the dependent variables), based on theoretical frameworks on affective determinants,^5^ and existing evidence.^7^, ^8^, ^23^

**RESEARCH IN CONTEXT**

1. **Systematic review**: Evidence shows apathy is a separate entity from depression, with distinct clinical profile and different neurobiology and potential treatments, which heralds a poorer prognosis. However, little research has examined apathy and depression separately in relation to health behaviors, despite their importance as modifiable risk factors for dementia. Our literature review using traditional sources failed to identify any studies of apathy, depression, and multiple health behaviors.
2. **Interpretation**: Apathy in older adults was associated with multiple health behaviors, and may be a target for health promotion, including interventions for dementia risk reduction. By contrast, depressive symptoms were not associated with health behaviors.
3. **Future directions**: Longitudinal analyses are needed to confirm directions of relationships. Other behavioral risk factors for dementia including sedentary behavior and sleep may also be associated with apathy. Mediation analyses could investigate potential behavioral pathways between apathy and incident dementia.

## 2 METHODS

### 2.1 Study design and participants

Individuals aged 70 to 90 in Sydney, New South Wales, Australia were invited to participate in a population‐based epidemiological cohort study, the Memory and Ageing Study (MAS),^28^ between 2005 and 2007. Inclusion required dwelling in a defined region, and sufficient ability in English for assessment. Exclusion criteria included diagnoses of dementia or neurodegenerative conditions among others; comprehensive description of the study's aims, sampling strategy, recruitment via compulsory electoral roll, and methodology is available in Sachdev et al.^28^ Written informed consent was obtained from participants (*n* = 1037) and their informants. Face‐to‐face assessments included neuropsychological and medical examinations and history. Psychosocial questionnaires were returned by post; blood or saliva was collected. Informants completed phone and postal questionnaires. Ethics approval was obtained from the University of New South Wales and South‐Eastern and Illawarra Area Health Service committees.

### 2.2 Measures

#### 2.2.1 Affective symptoms

Self‐report symptoms of apathy and depression over the past week were captured by the Geriatric Depression Scale‐15 (GDS).^29^ These 15 items purposively exclude “somatic” symptoms to avoid confounding with physical, mobility, and sensory impairments, and required yes/no responses. Two subscales were created, based on factor analysis in this cohort^30^ and meta‐analysis of international factor analyses.^31^ The GDS‐3A apathy subscale comprises 3 items (2: “dropped activities”; 9: “prefers to stay home”; 13: “feeling without energy” reverse scored) that load on a single factor and is formed by summing responses (range 0–3). This subscale has strong face validity in assessing apathy, although may have suboptimal reliability, due to its brevity.^15^, ^32^ The remaining 12 items formed a depression rating independent of apathy (GDS‐12D: range 0–12), covering symptoms of dysphoria, positive mood, and memory.^31^ Psychometric properties of the GDS‐12D have not been reported, although it is used consistently alongside the GDS‐3A. For primary analyses, continuous subscale scores of apathy and depression were used. To describe prevalence, cut‐off scores for presence of clinically relevant symptoms were also applied to each subscale (GDS‐3A ≥ 2; GDS‐12D ≥ 2). These are not yet validated against clinical diagnoses, with low sensitivity;^32^ however, are used for comparability to prior literature.^13^, ^17^, ^19^ An alternative GDS item 9 was administered in MAS (“I prefer to stay home *at night*, rather than going out and doing things”), as per Brink.^33^

Self‐report fatigue was derived from item 12 of the Assessment of Quality of Life‐6D (AQoL‐6D).^34^ Higher scores represented greater fatigue, after reverse scoring and recoding the Likert‐type responses (range 0–4). The AQoL‐6D has strong evidence of reliability and validity in measuring quality of life.^34^, ^35^ Its fatigue item has concurrent validity against more comprehensive measures of fatigue.^36^

Informant‐report apathy was from the Neuropsychiatric Inventory–apathy (NPI‐A).^37^ During telephone interview, if screening indicated presence of apathy, ratings of frequency (1–occasionally to 4–very frequently) and severity (1–mild to 3–severe) were made, and multiplied for the domain score used in primary analyses (range 0–12). Whilst widely used, the NPI‐A measure has evidence of inadequate psychometric properties, and susceptibility to zero inflation.^15^, ^38^ A cut‐off score validated in persons without dementia (≥ 1)^39^ determined prevalence.

#### 2.2.2 Health behaviors

Physical activity was a continuous measure of total minutes per week, weighted by intensity.^40^ Frequency and time spent on eight activities (bowls [lawn bowling], golf, swimming, dancing, bicycling, tennis, jogging, aerobics) were reported using items from UK Whitehall‐II.^41^ Planned walking for exercise and additional activities reported by participants were also recorded. Intensity was assigned to each activity, and used to weight time spent on each by multiplication: mild (1), moderate (2), or vigorous (3).

Dietary intake was a continuous measure of overall quality, adhering to Australian Dietary Guidelines, scored by the Dietary Guideline Index (DGI‐2013).^42^ Higher scores indicated better adherence to age‐/sex‐specific recommendations (range 0–90). Daily intake over the past year was reported via an 80‐item food frequency questionnaire.^43^ Photographs illustrated usual portion sizes.

Alcohol use was assessed via items from the Alcohol Use Disorders Identification Test.^44^ Participants reported frequency over the past year (never; monthly or less; 2–4× monthly; 2–3× weekly; 4–6× weekly; daily) and quantity of standard drinks typically consumed (1; 2–3; 4–5; 6–7; ≥8; modified for Australian standard drinks [10 g ethanol], of which illustrations were provided). For comparison to prior research, we recoded frequency as “weekly or more” and quantity as “2 or more drinks per day” binomially. Reports of lifetime abstinence and period of heavier consumption were also obtained.

Smoking was reported yes/no for current (the past month) and lifetime (regularly smoked in the past).

For the behavioral risk index, behaviors (physical activity, diet, alcohol, smoking) were recoded as 1 (at risk) or 0 (not at risk) based on current guidelines, and summed to give a score out of a possible 4.^45^ Physical inactivity risk was < 150 minutes of moderate‐to‐vigorous intensity activity weekly, as per Australian and international guidelines.^46^, ^47^ Participants with DGI‐2013 score in the lowest tertile were categorized at dietary risk, per previous literature.^45^ Risky alcohol use was > 10 standard drinks weekly, based on the 2020 Australian guidelines.^48^ Current smoking was considered a risk factor.^49^

#### 2.2.3 Other participant characteristics

Participants reported sex, education, non‐English‐speaking background (NESB), marital status, living situation, lifetime occupation, and vision impairment. Age was calculated (date of assessment–date of birth). Global cognition was assessed with Mini‐Mental State Examination.^50^ Indices of chronic conditions (cardiovascular, endocrine, urinary, respiratory, skeletal, circulatory, central nervous system conditions, and cancer) and cardiovascular risk (including smoking, diabetic status, systolic blood pressure, cholesterol and high‐density lipoprotein levels, antihypertensive medications) were computed, based on pre‐existing measures;^51^, ^52^ see Tables SA and SB in supporting information for details. Apolipoprotein E (*APOE*) Ɛ4 allele carriage was assessed by blood or saliva DNA analysis.

### 2.3 Analyses

Analyses were undertaken using SPSS 28. Assumptions including multicollinearity were evaluated. Categorical variables were dummy coded, and continuous scores mean corrected, to reduce multicollinearity for interactions. Descriptive analyses undertaken were *t* tests, *χ* ^2^, and Pearson correlations. Statistical significance was assumed at *P* < 0.05, due to the exploratory nature of analyses.

Primary analyses were eight hierarchical multiple regressions. For two continuous dependent variables (current physical activity, diet), two linear regressions were run. For six categorical dependent variables (alcohol: current frequency and quantity, lifetime abstinence, and past period of heavier consumption; smoking: current and lifetime), logistic regressions were run. Two apathy measures were available; both were analyzed for completeness, and because correlation analyses indicated lack of convergent validity (*r* = 0.09). Thus, two versions are reported for each analysis: Version A using the continuous self‐report apathy score, Version B the continuous informant‐report score.

Regression models were chosen a priori, with a prespecified hierarchical order of entry into the analysis to address the research questions, as per the following. Model 1 included apathy, the main independent variable of interest (either self‐ or informant‐report score). Model 1 also contained control variables, namely sociodemographics (sex, age, education, NESB, marital status, living situation, occupation), global cognition, vision impairment and *APOE* Ɛ4 carrier status; included as established correlates of health or health behaviors, as supported by correlations run in this sample (see Table SC in supporting information). Model 2 included scores for depression and fatigue, and Model 3 included their interaction terms with apathy (created by multiplying mean‐corrected apathy by depression scores, and apathy by fatigue scores), to explore whether they explained associations instead. An interaction term between sex and apathy (sex x apathy) was additionally included in Model 3. Finally, Model 4 included potential confounds, or mediators of the hypothesized associations. These were cardiovascular risk and chronic condition indices, collectively termed “health status,” which have established associations with apathy and health behaviors.

To quantify associations between affective symptoms and the behavioral risk index (an ordinal variable), multinomial regression analysis investigated apathy, fatigue, depression scores, sex, and their interactions as predictors of the risk index (categorized as none, one, two, three/four risks [collapsed due to low prevalence of all four behavioral risks]). This analysis controlled for all other covariates (sociodemographics other than sex, global cognition, vision impairment, *APOE* Ɛ4 carrier status).

## 3 RESULTS

### 3.1 Participant characteristics, affective symptoms, and health behaviors

Sample characteristics of 1037 community‐dwelling participants (mean 78 years; 55.2% women; 15.8% from non–English‐speaking backgrounds) are presented in Table 1. Sex differences were evident across sociodemographic but not clinical characteristics.

**TABLE 1:** Participant sociodemographic and clinical characteristics, affective symptoms, health behaviors, and informant characteristics for the Sydney Memory and Ageing Study cohort, and separately for men and women.

Characteristic	Total sample (*n* = 1,037)	Women (*n* = 572)	Men (*n* = 465)	Test of sex difference
**Participant sociodemographics**				
Age (years), mean (SD)	78.33 ± 4.81 (70–90)	78.39 ± 4.91 (70–90)	78.26 ± 4.69 (70–90)	*t* (1035) = −0.45, *P* = 0.66
Non‐English speaking background, % (*n*)	15.8 (164)	14.3 (82)	17.6 (82)	*χ* ^2^ = 2.10, *P* = 0.15
Education (years), mean (SD)	11.60 ± 3.47 (3–24)	11.03 ± 3.05 (6–24)	12.30 ± 3.83 (3–23)	*t* (1035) = 5.93, *P* < 0.001
Marital status (married/de facto), % (*n*)	41.4 (428)	38.1 (217)	45.2 (209)	*χ* ^2^ = 5.30, *P* = 0.021
Occupation (ASCO), mean (SD)	4.05 ± 2.59 (1–10)	5.06 ± 2.65 (1–10)	2.80 ± 1.87 (1–9)	*t* (1024) = −15.37, *P* < 0.001
Living situation (alone), % (*n*)	52.8 (544)	39.5 (225)	69.3 (319)	*χ* ^2^ = 91.16, *P* < 0.001
**Clinical characteristics**				
Global cognition (MMSE), mean (SD)	28.70 ± 1.34 (24–30)	28.76 ± 1.33 (24–30)	28.64 ± 1.36 (24–30)	*t* (1035) = −1.43, *P* = 0.153
*APOE* Ɛ4 carriage (present), % (*n*)	22.6 (222)	22.0 (118)	23.2 (104)	*χ* ^2^ = 0.20, *P* = 0.65
Vision impairment (present), % (*n*)	9.7 (100)	11.2 (64)	7.8 (36)	*χ* ^2^ = 3.52, *P* = 0.06
Cardiovascular risk index, mean (SD)	4.20 ± 3.13 (−5 to 16)	4.19 ± 3.48 (−5 to 16)	4.21 ± 2.64 (−3 to 10)	*t* (996) = .130, *P* = 0.897
Chronic condition index, mean (SD)	18.67 ± 9.00 (0–50)	19.43 ± 9.07 (0–45.45)	17.75 ± 8.83 (0–50)	*t* (1035) = −2.69, *P* = 0.007
**Affective symptoms**				
***Apathy***				
Self‐report (GDS‐3A)				
Magnitude (mean [SD])	1.49 ± 0.95 (0–3)	1.48 ± 0.95 (0–3)	1.50 ± 0.95 (0–3)	*t* (1028) = 0.27, *P* = 0.789
Prevalence (≥2, % [*n*])	48.9 (504)	49.5 (281)	48.3 (223)	*χ* ^2^ = 0.15, *P* = 0.701
Informant‐report (NPI‐apathy domain)				
Magnitude (mean Freq × Sev [SD])	0.06 ± 0.52 (0–12)	0.03 ± 0.31 (0–4)	0.10 ± 0.70 (0–12)	*t* (971) = 1.67, *P* = 0.095
Prevalence (≥1, % [*n*])	2.9 (28)	2.2 (12)	3.7 (16)	*χ* ^2^ = 1.93, *P* = 0.165
***Depression***				
Self‐report (GDS‐12D)				
Magnitude (mean [SD])	0.79 ± 1.60 (0–12)	0.68 ± 1.35 (0–12)	0.93 ± 1.85 (0–12)	*t* (1027) = 2.51, *P* = 0.012
Prevalence (≥2, % [*n*])	15.6 (162)	14.7 (83)	17.1 (79)	*χ* ^2^ = 1.10, *P* = 0.293
Informant‐report (NPI‐depression domain)				
Magnitude (mean Freq × Sev [SD])	0.27 ± 0.95 (0–12)	0.31 ± 1.08 (0–12)	0.23 ± 0.77 (0–6)	*t* (965) = −1.17, *P* = 0.241
Prevalence (≥1, % [*n*])	12.7 (123)	13.9 (75)	11.2 (48)	*χ* ^2^ = 1.57, *P* = 0.211
***Fatigue*** (AQoL‐6D item 12), mean (SD)	2.37 ± 0.79 (1–5)	2.48 ± 0.79 (1–5)	2.47 ± 0.78 (1–5)	*t* (995) = −0.14, *P* = 0.889
**Health behaviors**				
***Physical activity***				
Minutes/week (weighted by intensity), mean (SD)	454.80 ± 451.95 (2.30–4464.00)	378.28 ± 352.96 (2.30–2170.80)	542.70 ± 530.87 (6.90–4464.00)	*t* (894) = 5.52, *P* < 0.001
Risk factor present, % (*n*)	83.4 (836)	86.0 (478)	80.3 (358)	*χ* ^2^ = 5.82, *P* < 0.05
***Dietary intake***				
Quality (total DGI‐13), mean (SD)	43.78 ± 10.14 (12–73)	44.27 ± 10.20 (15–73)	43.17 ± 10.04 (12–73)	*t* (964) = −1.69, *P* = 0.092
Risk factor present, % (*n*)	36.5 (353)	35.1 (188)	38.4 (165)	*χ* ^2^ = 1.12, *P* = 0.290
***Alcohol consumption***				
Frequency (> 1/ week), % (*n*)	55.2 (572)	45.8 (262)	66.7 (310)	*χ* ^2^ = 45.14, *P* < 0.001
Quantity (≥2 standard drinks), % (*n*)	49.7 (515)	39.2 (224)	62.6 (291)	*χ* ^2^ = 55.90, *P* < 0.001
Lifetime abstinence, % (*n*)	5.6 (58)	7.9 (45)	2.8 (13)	*χ* ^2^ = 12.49, *P* < 0.001
Lifetime period of heavier consumption, % (*n*)	51.3 (529)	40.2 (228)	64.9 (301)	*χ* ^2^ = 62.11, *P* < 0.001
Risk factor present, % (*n*)	33.4 (346)	22.2 (127)	47.1 (219)	*χ* ^2^ = 71.18, *P* < 0.001
***Smoking***				
Current (risk factor present), % (*n*)	3.9 (40)	4.2 (24)	3.5 (16)	χ^2^ = 0.39, *P* = 0.533
Lifetime, % (*n*)	54.0 (559)	42.7 (244)	67.9 (315)	*χ* ^2^ = 65.22, *P* < 0.001
***Total number risk factors*** present, % (*n*)				
Zero risks	7.0 (66)	7.0 (37)	6.9 (29)	*χ* ^2^ = .005, *P* = 0.943
One risk	44.3 (420)	50.9 (269)	35.9 (151)	*χ* ^2^ = 21.59, *P* < 0.001
Two risks	34.0 (323)	30.7 (162)	38.2 (161)	*χ* ^2^ = 5.96, *P* = 0.015
Three risks	13.8 (131)	10.4 (55)	18.1 (76)	*χ* ^2^ = 11.48, *P* < 0.001
Four risks	0.9 (9)	0.9 (5)	1.0 (4)	*χ* ^2^ = 0.00, *P* = 0.996
**Informant (*n* = 999)**				
Age (years), mean (SD)	62.73 (14.05)	61.81 (14.53)	63.87 (13.36)	*t* (997) = 2.31, *P* = 0.021
Female sex, % (*n*)	68.7 (693)	64.5 (361)	73.9 (332)	*χ* ^2^ = 10.41 *P* = 0.001
Living with participant, % (*n*)	27.8 (288)	18.4 (105)	39.4 (183)	*χ* ^2^ = 57.80, *P* < 0.001
Relationship to participant, % (*n*)				
Spouse	28.4 (229)	16.8 (73)	41.9 (156)	*χ* ^2^ = 62.12, *P* < 0.001
Child	36.6 (295)	45.2 (196)	26.6 (99)	*χ* ^2^ = 29.70, *P* < 0.001
Relative (sibling, grandchild, other)	10.0 (81)	12.7 (55)	7.0 (26)	*χ* ^2^ = 7.16, *P* = 0.007
Other (friend, other)	24.9 (201)	25.3 (110)	24.5 (91)	*χ* ^2^ = 0.08, *P* = 0.773

Abbreviations: *APOE*, apolipoprotein E; AQoL‐6D, Assessment of Quality of Life‐6D; ASCO, Australian Standard Classification of Occupations; DGI‐13, Dietary Guideline Index‐2013; GDS, Geriatric Depression Scale; MMSE, Mini‐Mental State Examination; NPI, Neuropsychiatric Inventory; SD, standard deviation.

Prevalence of affective symptoms varied between perspectives, from 48.9% for apathy and 15.6% for depression via self‐report, to 2.9% and 12.7%, respectively, based on informant report. There were no sex differences in these symptoms.

Engagement in health behaviors was poor, with only 7.0% of participants having no behavioral risk factors (physical inactivity, poorer diet, risky alcohol use, or smoking), based on current guidelines. Physical inactivity had highest prevalence (83.4%) and was more common in women; other risks were more frequent in men.

### 3.2 Affective predictors of individual health behaviors

An identical hierarchical approach was taken for each regression analysis , guided by the research questions. The first model addressed the primary question on whether apathy predicted the health behavior, controlling for covariates. Subsequent models added, in turn, depression and fatigue scores, interaction terms, and health status variables, to check if associations found for apathy were independent of these constructs.

#### 3.2.1 Physical activity

The first model in Table 2A showed self‐report apathy was associated with reduced physical activity, explaining 6.7% of its variance, over and above control variables (sociodemographics and visual, cognitive, and genetic factors), consistent with a medium effect size. The second model indicated this association did not change when depression and fatigue scores were included in the regression equation. The third and fourth models also demonstrated no attenuation of the association when interaction terms were included in the model, nor health status. Additional results were that depression was not independently associated with physical activity, whereas fatigue had a small to medium effect size for reduced physical activity.

**TABLE 2:** Linear regression models predicting physical activity in the Sydney Memory and Ageing Study cohort.

	A. Self‐report apathy (*n* = 800)					B. Informant‐report apathy (*n* = 806)				
	*ΔR^2^*	*ΔF*	*B*	*β*	*t*	*ΔR^2^*	*ΔF*	*B*	*β*	*t*
Model 1: Apathy	0.067	59.46***				0.003	2.11			
Self‐report (GDS‐3A)			–129.07	–0.27	–7.71***			N/A	N/A	N/A
Informant report (NPI)			N/A	N/A	N/A			–43.17	–0.05	–1.45
Model 2: Depression & fatigue	0.017	7.62***				0.049	20.52***			
Self‐report depression (GDS‐12D)			11.00	0.04	1.04			4.52	0.02	0.40
Self‐report fatigue (AQoL‐6D)			–91.25	–0.16	–3.89***			–138.00	–0.24	–6.31***
Model 3: Interactions	0.007	2.28				0.001	0.41			
Apathy × depression			13.99	0.05	1.24			1.08	0.00	0.06
Apathy × fatigue			22.19	0.03	0.92			46.16	0.16	0.64
Apathy × sex			–64.05	–0.09	–1.97*			‐89.76	1.10	–0.95
Model 4: Health status	0.006	2.74				0.009	3.83*			
CVD risk index			–7.59	–0.05	–1.54			–9.12	–0.06	–1.78
Chronic condition index			–2.98	–0.06	–1.60			–3.67	0.07	–1.90

*Note. B* = Unstandardized regression coefficient. *Beta* = *β* = Standardized regression coefficient. Model 1 included sex, age, non‐English speaking background, education, marital status, living situation, occupation, global cognition, *APOE* Ɛ4 allele carriage, and vision impairment. Model 1 also included apathy (self‐report part A, informant‐report part B). Model 2 included depression and fatigue. Model 3 included interactions: apathy x depression, apathy × fatigue, and apathy × sex. Model 4 included CVD risk index and chronic condition index.

Abbreviations: *APOE*, apolipoprotein E; AQoL‐6D, Assessment of Quality of Life‐6D; CVD, cardiovascular disease; GDS, Geriatric Depression Scale; NPI, Neuropsychiatric Inventory.

***P < 0.001, two‐tailed. **P < 0.01, two‐tailed. *P < 0.05, two‐tailed.

In comparison, Table 2B shows informant‐report apathy was not associated with physical activity. Other results remained the same in this alternative analysis, although there was evidence of an increase in variance explained by depression and fatigue.

#### 3.2.2 Dietary intake

As shown in Tables 3A and 3B, using the same hierarchical approach, diet quality was not associated with apathy measures (Model 1), although both self‐ and informant report approached significance. Additionally, depressive symptoms were not associated with diet quality, whereas fatigue independently predicted poorer diet quality in one analysis (Table 3B, Model 2), but this association was lost after final adjustment for health status.

**TABLE 3:** Linear regression models predicting dietary intake in the Sydney Memory and Ageing Study cohort.

	A. Self‐report apathy (*n* = 856)					B. Informant‐report apathy (*n* = 820)				
	*ΔR^2^*	*ΔF*	*B*	*β*	*t*	*ΔR^2^*	*ΔF*	*B*	*β*	*t*
**Model 1: Apathy**	0.004	3.27				0.004	3.08			
Self‐report (GDS‐3A)			–0.68	–0.06	–1.81			N/A	N/A	N/A
Informant report (NPI)			N/A	N/A	N/A			–1.76	–0.06	–1.76
**Model 2: Depression & fatigue**	0.007	3.24*				0.010	4.26*			
Self‐report depression (GDS‐12D)			–0.35	–0.05	–1.47			–0.31	–0.05	–1.23
Self‐report fatigue (AQoL‐6D)			–0.91	–0.07	–1.72			–1.07	–0.08	–2.22*
**Model 3: Interactions**	0.002	0.55				0.010	0.05			
Apathy × depression			–0.08	–0.01	–0.33			–0.05	–0.01	–0.17
Apathy × fatigue			–0.39	–0.03	–0.73			–0.03	–0.01	–0.12
Apathy × sex			–0.59	–0.04	–0.81			–0.10	–0.01	–0.14
**Model 4: Health status**	0.001	0.58				0.001	0.60			
CVD risk index			–0.01	–0.00	–0.09			–0.01	–0.00	–0.08
Chronic condition index			0.05	0.04	1.08			0.05	0.04	1.07

*Note. B* = Unstandardized regression coefficient. *Beta* = *β* = Standardized regression coefficient. Model 1 included sex, age, non‐English speaking background, education, marital status, living situation, occupation, global cognition, *APOE* Ɛ4 allele carriage, and vision impairment. Model 1 also included apathy (self‐report part A, informant‐report part B). Model 2 included depression and fatigue. Model 3 included interactions: apathy × depression, apathy × fatigue, and apathy × sex. Model 4 included CVD risk index and chronic condition index.

Abbreviations: *APOE*, apolipoprotein E; AQoL‐6D, Assessment of Quality of Life‐6D; CVD, cardiovascular disease; GDS, Geriatric Depression Scale; NPI, Neuropsychiatric Inventory.

***P < 0.001, two‐tailed. **P < 0.01, two‐tailed. *P < 0.05, two‐tailed.

#### 3.2.3 Alcohol use

Self‐report apathy was inversely associated with both frequency and quantity of current alcohol use (Model 1 in Tables 4, 5). That is, apathy explained up to 7% of variance, predicting lower odds of frequent consumption (at least weekly), and lower odds of higher quantity (consuming two or more drinks, compared to one). These findings were of medium effect size, and not attenuated by inclusion of other variables in Models 2 through 4. In contrast, fatigue was independently associated with higher quantity but not frequency (Model 2), whereas informant‐report apathy (Model 1 in Tables 4, 5) and depression (Model 2) had no associations with current alcohol use. Tables SD and SE in supporting information show additional analyses for lifetime alcohol use, with results indicating lifetime abstinence and past period of heavier consumption held no relationship with any of the affective symptoms.

**TABLE 4:** Logistic regression models predicting more frequent alcohol consumption in the Sydney Memory and Ageing Study cohort.

	A. Self‐report apathy (*n* = 897)			B. Informant‐report apathy (*n* = 857)		
	ΔX2	OR	95% CI	ΔX2	OR	95% CI
**Model 1: Apathy**	6.78**			1.32		
Self‐report (GDS‐3A)		0.82	0.70, 0.95**		N/A	N/A
Informant report (NPI)		N/A	N/A		0.84	0.61, 1.17
**Model 2: Depression & fatigue**	0.49			0.46		
Self‐report depression (GDS‐12D)		1.00	0.91, 1.10		0.99	0.89, 1.10
Self‐report fatigue (AQoL‐6D)		1.08	0.87, 1.34		0.94	0.78, 1.15
**Model 3: Interactions**	2.74			1.69		
Apathy × depression		0.99	0.89, 1.10		1.06	0.87, 1.30
Apathy × fatigue		0.85	0.69. 1.06		1.32	0.68, 2.57
Apathy × sex		1.07	0.79, 1.44		1.67	0.58, 4.81
**Model 4: Health status**	2.13			2.47		
CVD risk index		0.97	0.93, 1.02		0.98	0.93, 1.02
Chronic condition index		1.00	0.98, 1.01		0.99	0.97, 1.01

*Note*. Dependent variable reference group were those who reported consuming alcohol weekly or less often. Model 1 included sex, age, non‐English speaking background, education, marital status, living situation, occupation, global cognition, *APOE* Ɛ4 allele carriage, and vision impairment. Model 1 also included apathy (self‐report part A, informant‐report part B). Model 2 included depression and fatigue. Model 3 included interactions: apathy × depression, apathy × fatigue, and apathy × sex. Model 4 included CVD risk index and chronic condition index.

Abbreviations: *APOE*, apolipoprotein E; AQoL‐6D, Assessment of Quality of Life‐6D; C,I confidence interval; CVD, cardiovascular disease; GDS, Geriatric Depression Scale; NPI, Neuropsychiatric Inventory; OR, odds ratio.

***P < 0.001, two‐tailed. **P < 0.01, two‐tailed. *P < 0.05, two‐tailed.

**TABLE 5:** Logistic regression models predicting higher quantity of alcohol consumption in the Sydney Memory and Ageing Study cohort.

	A. Self‐report apathy (*n* = 896)			B. Informant‐report apathy (*n* = 857)		
	*ΔX2*	*OR*	*95% CI*	*ΔX2*	*OR*	*95% CI*
**Model 1: Apathy**	4.06*			2.64		
Self‐report (GDS‐3A)		0.86	0.73, 0.99*		N/A	N/A
Informant report (NPI)		N/A	N/A		0.74	0.49, 1.12
**Model 2: Depression & fatigue**	6.29*			2.04		
Self‐report depression (GDS‐12D)		0.95	0.86, 1.04		0.97	0.87, 1.07
Self‐report fatigue (AQoL‐6D)		1.31	1.05, 1.63*		1.15	0.94, 1.41
**Model 3: Interactions**	2.14			3.56		
Apathy × depression		0.95	0.86, 1.05		0.83	0.59, 1.16
Apathy × fatigue		0.94	0.75, 1.17		1.63	0.77, 3.46
Apathy × sex		0.96	0.71, 1.30		1.14	0.37, 3.51
**Model 4: Health status**	1.86			3.04		
CVD risk index		1.01	0.96, 1.06		1.01	0.97, 1.06
Chronic condition index		0.99	0.97, 1.01		0.99	0.97, 1.00

*Note*. Dependent variable reference group are those who reported consuming no more than one standard drink on a typical drinking day. Model 1 included sex, age, non‐English speaking background, education, marital status, living situation, occupation, global cognition, *APOE* Ɛ4 allele carriage, and vision impairment. Model 1 also included apathy (self‐report part A, informant‐report part B). Model 2 included depression and fatigue. Model 3 included interactions: apathy x depression, apathy x fatigue, and apathy x sex. Model 4 included CVD risk index and chronic condition index.

Abbreviations: *APOE*, apolipoprotein E; AQoL‐6D, Assessment of Quality of Life‐6D; CI, confidence interval; CVD, cardiovascular disease; GDS, Geriatric Depression Scale; NPI, Neuropsychiatric Inventory; OR, odds ratio.

***P < 0.001, two‐tailed. **P < 0.01, two‐tailed. *P < 0.05, two‐tailed.

#### 3.2.4 Smoking

No evidence of relationships between current or lifetime smoking with apathy measures was found (Model 1, Tables SF, SG in supporting information), nor with depression (Model 2). Fatigue predicted current smoking (Model 2), but this was attenuated by final adjustment for health status.

### 3.3 Affective predictors of multiple health behaviors

Self‐report apathy was significantly associated with the behavioral risk index (*P* = 0.003), as shown in a multinomial regression analysis (Table 6). Individuals reporting greater apathy were significantly more likely to have one or multiple risk factors. Specifically, odds of having one, two, or three/four risk factors (compared to zero) increased by 97%, 60%, and 62%, respectively, with greater apathy, controlling for a comprehensive range of confounds.

**TABLE 6:** Multinomial regression models predicting number of behavioral risk factors in Sydney Memory and Ageing Study participants (*n* = 854; reference group with zero risk factors).

	Model		Zero risk			One risk				Two risks				Three or four risks		
	*χ* ^2^	*P*	*M (n)*	*SD (%)*	*M (n)*	*SD (%)*	*OR*	*95% CI*	*M (n)*	*SD (%)*	*OR*	*95% CI*	*M (n)*	*SD (%)*	*OR*	*95% CI*
**Model 1: Affective symptoms**																
Apathy symptoms (GDS‐3A)	13.77	0.003	0.98	0.93	1.54	0.94	1.97	1.33, 2.92	1.47	0.94	1.60	1.08, 2.39	1.61	0.95	1.62	1.05, 2.50
Depressive symptoms (GDS‐12D)	10.11	0.018	0.77	1.31	0.63	1.36	0.79	0.65, 0.97	0.72	1.56	0.87	0.72, 1.05	1.23	2.01	0.96	0.79, 1.17
Fatigue (AQoL‐6D item 12)	9.52	0.023	2.10	0.72	2.45	0.75	1.60	0.99, 2.61	2.49	0.81	1.77	1.09, 2.89	2.64	0.80	2.21	1.30, 3.75
Sex (females, reference group)	22.00	<0.001	(37)	(7.0)	(269)	(50.9)			(162)	(30.7)			(60)	(11.4)		
Males			(29)	(6.9)	(151)	(35.9)	0.57	0.28, 1.20	(161)	(38.2)	1.22	0.58, 2.57	(80)	(19.0)	1.44	0.64, 3.26
**Model 2: As above, with interaction effects**																
Apathy × sex interaction	6.14	0.105	–0.13	0.68	0.03	0.57	0.46	0.23, 0.90	–0.03	0.66	0.45	0.23, 0.89	0.09	0.70	0.48	0.23, 1.01
Apathy × depression interaction	2.44	0.487	0.68	1.23	0.31	1.39	0.97	0.79, 1.19	0.34	1.58	0.95	0.78, 1.16	0.37	1.94	0.90	0.74, 1.09
Apathy × fatigue interaction	0.40	0.939	0.43	0.68	0.35	0.66	0.94	0.57, 1.55	0.38	0.74	0.98	0.59, 1.62	0.42	0.75	1.03	0.60, 1.76

*Note*. Dependent variable reference group are those with zero behavioral risk factors. Independent variable reference groups are in parentheses. Model 1 included self‐report apathy, depression, fatigue, sex, age, non‐English speaking background, education, marital status, living situation, occupation, global cognition, *APOE* Ɛ4 allele carriage, vision impairment, CVD risk index, and chronic condition index. Model 2 additionally included interactions: apathy × depression, apathy × fatigue, and apathy × sex.

Abbreviations: *APOE*, apolipoprotein E; AQoL‐6D, Assessment of Quality of Life‐6D; CI, confidence interval; CVD, cardiovascular disease; GDS, Geriatric Depression Scale; NPI, Neuropsychiatric Inventory; OR, odds ratio; SD, standard deviation.

In the same analysis, fatigue and depressive symptoms were also associated with the behavioral risk index, but in opposite directions. Compared to zero risks, odds of one risk factor were 22% lower with increased depressive symptomatology whereas greater fatigue was associated with higher odds of two, or three/four factors, by 77% and 122%, respectively.

### 3.4 Moderation effects

Interactions for apathy with depression, fatigue, and sex were examined in all analyses (Tables 2, 3, 4, 5 [Model 3] and Table 6 [Model 2]). Significant moderation by sex was found in analyses of physical activity and the behavioral risk index, with the effect of apathy more pronounced for male participants.

## 4 DISCUSSION

To our knowledge, this is the first study to investigate affective symptoms of apathy, depression, and fatigue as predictors of multiple health behaviors. Among symptom measures, only self‐report apathy held a broad range of associations with health behaviors, as hypothesized. These associations were robust to confounding, independent of depression and fatigue, and in alignment with prior research and current conceptualizations of apathy. That apathy is defined as quantitative reduction in goal‐directed behavior^12^ was reflected in reduction in all four health behaviors, irrespective of their nature as risky or protective for health. This consistency in evidence was unique to self‐report apathy and suggests the relevance of this measure for health, contrasting with depression, fatigue, and informant‐report apathy, which had nil or inconsistent associations with health behaviors.

Lifestyle risk factors were highly prevalent in this cohort, and commensurate with Australian population estimates,^47^, ^49^, ^53^ although smoking was less common,^49^ potentially due to higher education and/or socioeconomic advantage in MAS participants. Almost half of the sample (48.9%) had significant apathy on self‐report, higher than previous reports,^13^ and pooled prevalence of 31.1% from 15 international cohorts.^19^ This may relate to MAS participants’ age ranging up to 90, as apathy was seen to increase chronologically, up to 41% at 90 years in the Leiden‐85+ study.^54^ Alternatively, the observed prevalence may have been inflated, given previous reports of low sensitivity of this measure,^32^ and one slightly modified item in MAS.

Addressing analyses between apathy and individual health behaviors in turn, first, physical activity was strongly associated with lower self‐report apathy, confirming prior research with self‐report^22^, ^23^ and objective measures of activity.^22^, ^24^ Each additional apathy symptom explained 2 hours less physical activity per week (weighted by intensity), a substantial effect, which was more pronounced in male participants. Second, dietary intake was examined in conjunction with apathy for the first time in a community cohort. While neither association with self‐ nor informant‐report apathy was significant, their direction suggested poorer diet quality may relate to apathy. This was consistent with our finding for physical activity, given evidence that these two “health‐promoting” behaviors cluster together,^21^ but remains to be confirmed.

Third, self‐report apathy was associated with reduced current alcohol consumption, and fourth, not associated with smoking. These findings mostly align with prior research in community‐dwelling older adults.^23^, ^25^, ^26^ Regarding alcohol, we replicate evidence of these GDS apathy items relating to lower quantity. However, lower frequency had been previously linked with only one item (“feeling without energy”), whereas the other two items were associated with higher frequency.^25^

Our study extends knowledge by showing no associations between past health‐risk behaviors and current apathy. This was surprising, as smoking and alcohol across the lifespan lead to poorer cardiovascular outcomes, and cardiovascular pathologies cause apathy (the vascular apathy hypothesis).^55^ Apathy was associated with greater behavioral risk in males, indicating a potential sex difference that requires further investigation. Overall, results suggest a nuanced association between apathetic symptoms and different aspects of health‐risk and ‐promoting behaviors.

After pooling multiple health behaviors into a combined risk index, only apathy was associated with greater odds of one or multiple risk factors (compared to zero), independent of confounding factors. This striking result supports an argument for a meaningful relationship between self‐report apathy and health behaviors, especially as combining multiple behaviors into a single score may better reflect relations between risk factors in real‐life contexts.^27^ However, replication of this novel finding is required.

By contrast, informant‐report apathy yielded low prevalence—compared to self‐report as well as informant report in other cohorts^14^—and was not associated with any health behavior. The difference seen in results for the two apathy measures is notable, and could be considered a limitation, but was not unexpected. Increasingly, self‐ and informant‐report neuropsychiatric symptom measures are seen to perform differently in cognitively normal populations, and do not capture the same groups.^56^ Supporting this, in our sample the two correlated poorly, indicating lack of convergent validity. This may reflect source–method bias. While the recommendation is to assess apathy from the informant perspective for persons with dementia,^12^ this approach may not be optimal for persons without, and indeed has not been validated. Psychometrically, evidence of inadequate content and construct validity for both measures suggests they may not assess the exact same construct.^15^ In support, fatigue and depression together explained more variance in physical activity and diet, when informant‐report apathy was included in the model (compared to self‐report), suggesting this measure had less shared variance with overlapping constructs.

Regarding depression, the GDS‐12D used here can be considered a homogenous measure of dysphoric symptoms including low mood and affect, worthlessness, hopelessness, and not wanting to be alive. Unexpectedly, it was not associated with any individual health behavior, and *protective* against one risk factor. These findings contradict substantial literature on the salience of depression for health behaviors. For instance, physical activity is considered both an important protective factor against, and a treatment for, depressive disorders.^57^ We propose that effects found for depression in previous work may have been driven by apathy or other symptoms, which are often subsumed in depression scales.^9^ Supporting this, and consistent with our results, the few studies which separately analyzed apathy and depressive symptoms (as per the GDS‐3A and GDS‐12D) found the latter unrelated to physical activity.^23^, ^24^ Alternatively, controlling for comprehensive sociodemographic variables in our analyses may have attenuated the effect of depression.

### 4.1 Implications

These findings underscore the importance of apathy as a separate entity from depression.^10^ This is relatively well understood in psychogeriatrics, where apathy is considered a stand‐alone syndrome, with distinct clinical profile, neurobiology, and treatment, which heralds a poorer prognosis.^10^, ^11^ We extend this knowledge, by showing differential relations with multiple health behaviors. Apathy appeared to be a risk factor, whereas depression was potentially protective, although this direction of relationship remains to be confirmed with longitudinal data. This has implications across multiple fields of research in which health behaviors are key constructs.

First, apathy has received little attention within health behavior science. We propose that in older adults, apathy may be a determinant of health behaviors and a target for interventions. While ours may be one of the first quantitative reports, it is supported by substantial qualitative evidence on apathy as a barrier for healthy lifestyle activities, particularly physical activity.^20^ For instance, lived experience in neurological diseases indicated difficulties in initiating physical activity (behavioral apathy), and in identifying goals for action (cognitive apathy).^58^ In fact, affective constructs such as emotions, symptoms, and “feeling states” (including apathy) have consistently been the most predominant barriers to health behaviors identified in the literature, across populations.^8^ Despite this, they have been largely overlooked to date in designing behavior change interventions.^3^ Their inclusion is warranted, will meet important unmet needs, and should be considered, based on our findings and emerging theoretical frameworks on affective determinants.^5^

Importantly, apathy may help address two specific gaps within this field. First, maintenance of health behavior change is an ongoing challenge for researchers and the population alike.^2^ Lapses and relapses are common; behavioral change is rarely sustained over time.^2^, ^59^ Apathy is the most persistent neuropsychiatric symptom.^60^ This implies poorer engagement in health behaviors may be maintained long term, if underpinned by apathy as suggested by our findings. While this remains to be confirmed longitudinally, it is supported by apathy predicting dropout from a trial of a multidomain dementia prevention intervention.^61^ The second gap regards multiple health behaviors. Determining factors (such as apathy) which predict multiple risks may help optimize multiple behavior change interventions.^21^ These do not have a strong evidence base, yet their potential public health impact is high due to likely synergistic effects between behaviors,^27^, ^45^ exponentially increasing risk of diseases, including dementia.^62^

Second, within the dementia field, apathy is not established as an independent risk factor^1^ and has not been considered to date in risk reduction efforts, such as multidomain interventions. However, emerging “precision prevention” approaches suggest tailoring risk reduction to individual characteristics.^63^ Our findings demonstrate apathy is a characteristic linked to engagement with key components of multidomain interventions—physical activity in particular. Treatments for apathy may be able to be integrated into future individualized dementia risk reduction. For instance, behavioral activation holds promise for treating apathy,^64^ and is efficacious in promoting health behaviors.^65^ Psychostimulants improve apathy,^66^ and health outcomes including body mass index, potentially via increasing physical activity.^67^

Finally, this study contributes to ongoing efforts to better characterize apathy, and more broadly, mild behavioral impairment (MBI),^6^ which proposes persistent neuropsychiatric symptoms, particularly apathy, emerging in late life represent early manifestations of dementia. One current concern with the definition of apathy is that it is difficult to differentiate from fatigue.^68^ Both are multidimensional constructs, characterized by reduced goal‐directed behavior; this has been demonstrated experimentally as lower willingness to engage in physically effortful activity.^69^ Our results hint at partial overlap between apathy and fatigue, via somewhat similar patterns of associations with health behaviors. However, there was no evidence of interaction effects or confounding.

### 4.2 Strengths and limitations

Strengths included the large well‐characterized community‐based sample and comprehensive covariates. Limitations included lack of neurobiological and psychotropic medication covariates. We report cross‐sectional data from which directionality or causation cannot be determined; longitudinal evaluation is required, which may find bidirectional relationships or reverse causality. However, while literature does suggest a protective nature of behaviors such as physical activity against depression (i.e., reverse causation), no evidence for this was found in older adults, when both directions were examined longitudinally.^7^ Instead, supporting our hypothesis, depressive symptoms were a barrier to subsequent engagement in physical activity.

Generalizability was reduced by the relatively advantaged urban setting, exclusion of non‐English speakers and primarily White participants. Similar findings in a culturally diverse cohort of older Australians suggests applicability across contexts.^70^ Nonetheless, more epidemiological research is needed in under‐represented communities in Australia and globally, including indigenous populations and those living in rural and remote areas.

Subjective measures of health behaviors and affective symptoms can be biased and/or confounded. A strength of this research was that two apathy measures available from the MAS dataset were analyzed, to compare their results and avoid selective reporting bias. Nonetheless, replication using more objective tools, such as accelerometry, would support the clinical relevance of our findings, particularly as both apathy measures have evidence of suboptimal psychometric properties,^15^, ^32^ potentially influencing results. While the GDS‐3A derives from the preferred self‐report depression scale for older adults, with strong evidence of a single apathy factor supporting its use, its reported low sensitivity and reliability suggests potential misclassification bias toward the null.^32^ NPI scores tend to be zero‐inflated among other issues,^15^, ^38^ potentially biasing informant‐report apathy results. These measurement issues likely resulted in lack of power, which along with the exploratory nature of analyses meant we chose not to correct for multiple comparisons. Thus, significant findings may represent Type 1 error, particularly given the number of analyses. We also reported effect sizes to aid interpretation. Finally, single‐item measures (here, fatigue and smoking) are well validated, but do not assess multidimensional aspects of the construct.

## 5 CONCLUSION

Apathy was associated with physical inactivity, less alcohol use, and one or multiple behavioral risk factors. No or inconsistent relations were found between depressive or fatigue symptoms and individual health behaviors. Findings indicate the significance of apathy for multiple health behaviors and the necessity of distinguishing apathy from comorbid symptoms. Apathy should be considered when designing health promotion activities for older adults, including interventions for dementia risk reduction.

## AUTHOR CONTRIBUTIONS

Fleur Harrison: funding acquisition, conceptualization, methodology, data curation, formal analysis, writing—original draft, writing—reviewing and editing, visualization. Henry Brodaty: supervision, project administration, funding acquisition, data curation, writing—reviewing and editing. Moyra Mortby: supervision, writing—reviewing and editing. Karen Mather and Perminder Sachdev: funding acquisition, data curation, writing—reviewing and editing.

## CONFLICT OF INTEREST STATEMENT

Henry Brodaty is or has been an advisory board member or consultant to Biogen, Eisai, Eli Lilly, Roche, and Skin2Neuron. He is a Medical/Clinical Advisory Board member for Montefiore Homes and Cranbrook Care. Perminder S. Sachdev was on the expert advisory panel for Biogen and Roche Australia in 2020 and 2021. The other authors report no conflicts of interest. Author disclosures are available in the supporting information.

## CONSENT STATEMENT

All human subjects provided informed consent.

## Supporting information

Supporting Information

Supporting Information
