# Supplementary material for: Apathy as a determinant of health behaviors in older adults: Implications for dementia risk reduction
Source: Alzheimers Dement (Amst). 2023 Nov 23;15(4):e12505. doi: 10.1002/dad2.12505 (PMC10668002; doi:10.1002/dad2.12505)
Supplement: Supplementary file 1 — Supporting Information [file DAD2-15-e12505-s001.docx]

**SUPPLEMENTARY TABLE A. Variables included in the MAS Chronic Conditions Index, the body systems to which they belong and the cut-off points that define a deficit for that variable.**

| **Body System** | **Name** |  | **Deficit Definition** |
| --- | --- | --- | --- |
| Cardiovascular | Stroke |  | Yes = 1, No = 0 |
| Cardiovascular | TIA (mini-stroke) |  | Yes = 1, No = 0 |
| Cardiovascular | Problems with heart (diagnosed) |  | Yes = 1, No = 0 |
| Cardiovascular | High blood pressure |  | Yes = 1, No = 0 |
| Endocrine | Thyroid Disorder |  | Yes = 1, No = 0 |
| Urinary | Kidney Disease |  | Yes = 1, No = 0 |
| Urinary | Urinary Tract Infections |  | Yes = 1, No = 0 |
| Respiratory | Asthma |  | Yes = 1, No = 0 |
| Respiratory | Emphysema |  | Yes = 1, No = 0 |
| Respiratory | Chronic Obstructive Pulmonary Disease |  | Yes = 1, No = 0 |
| Respiratory | Bronchitis |  | Yes = 1, No = 0 |
| Skeletal | Arthritis |  | Yes = 1, No = 0 |
| Skeletal | Osteoporosis |  | Yes = 1, No = 0 |
| Circulatory | Claudication |  | Yes = 1, No = 0 |
|  | Anaemia |  | Yes = 1, No = 0 |
|  | Vitamin Deficiency |  | Yes = 1, No = 0 |
| Various | Cancer |  | Yes = 1, No = 0 |
| CNS | Head Injury |  | Yes = 1, No = 0 |
| CNS | Parkinsons's disease |  | Yes = 1, No = 0 |
| CNS | Epilepsy |  | Yes = 1, No = 0 |
|  | Number of recent falls |  | Yes = 1, No = 0 |
| Medications | Total # prescription meds (excludes vitamins, minerals, herbal) |  | >2 = 1, <= 2 = 0 |

The MAS Chronic Conditions Index measures of the number of deficits accumulated by an individual. It has been developed following the standard procedure for creating a frailty index, as developed by Searle et al. (Searle et al., 2008). For each participant, the index is calculated by dividing the number of deficits for a participant by the total number of deficits. Variables have been included which satisfy the following criteria:

1. The deficit must be associated with health status and must be acquired.
2. The prevalence of the deficit must increase with age.
3. The deficit must not saturate too early: i.e. it should not be ubiquitous in the community at too early an age.
4. The deficits selected must cover a range of systems.
5. The deficits that make up the index should be the same from one wave to the next if the index is to be used across waves.

**SUPPLEMENTARY TABLE B. Variables included in the MAS Cardiovascular Risk Index, the body systems to which they belong, the source of the data and further information.**

| **Body System** | **Name** | **Data Source** | **Definition** |
| --- | --- | --- | --- |
| Sociodemographics | Gender | Self-reported | Separate regression models run for males and females |
| Endocrine | Diabetes | Self-reported | Yes = 1, No = 0 |
| Cardiovascular | Systolic blood pressure | Medical examination | Average of 2 seated readings (unless only 1 reading had been done, then this value was used) |
| Cardiovascular | Total cholesterol level | Blood analysis | - |
| Cardiovascular | High-density lipoprotein (HDL) | Blood analysis | - |
| Overall health | Smoking status | Self-reported | Current = 1, abstainer = 0 |
| Overall health | Body mass index (BMI) | Medical examination | Only used where cholesterol or HDL data from blood analysis was unavailable |
| Medications | Current antihypertensive agent | Self-reported and/or presence on medication list | Participants do NOT receive factor scores based on whether or not they are currently taking antihypertensive medication. Instead, systolic blood pressure is rated differentially, based on whether or not the participant is taking antihypertensive medication (see reference for details). |

The CV Risk Index is a variable computed based on the research of the Framingham Stroke Study (http://www.framinghamheartstudy.org/index.html). Specifically, our index is based on the 10-year risk prediction of general cardiovascular disease (http://www.framinghamheartstudy.org/risk/gencardio.html) which is reported in (D’Agostino et al., 2008). Essentially, participants are rated on each of the factors described (age, cholesterol, etc) and assigned points for that factor. An individual’s CV risk score is simply the tally of the points they receive on each factor.

**References**

D’Agostino, R. B., Vasan, R. S., Pencina, M. J., Wolf, P. A., Cobain, M., Massaro, J. M., & Kannel, W. B. (2008). General Cardiovascular Risk Profile for Use in Primary Care. *Circulation*, *117*(6), 743–753. https://doi.org/10.1161/CIRCULATIONAHA.107.699579

Searle, S. D., Mitnitski, A., Gahbauer, E. A., Gill, T. M., & Rockwood, K. (2008). A standard procedure for creating a frailty index. *BMC Geriatrics*, *8*(1), 1–10. https://doi.org/10.1186/1471-2318-8-24/TABLES/3

**SUPPLEMENTARY TABLE C. Univariate correlations between apathy measures and participant characteristics**

| Measure |  | 1 | 2 | 3 | 4 | 5 | 6 | 7 | 8 |
| --- | --- | --- | --- | --- | --- | --- | --- | --- | --- |
|  |  |  |  |  |  |  |  |  |  |
| 1. GDS-3A (self-report apathy) |  | – |  |  |  |  |  |  |  |
| 2. NPI-G (informant-report apathy) |  | .09** | – |  |  |  |  |  |  |
| 3. Age (years) |  | .21** | .04 | – |  |  |  |  |  |
| 4. Education (years) |  | -.10** | .02 | -.06 | – |  |  |  |  |
| 5. Occupation (years) |  | .06 | -.04 | .08* | -.49** | – |  |  |  |
| 6. MMSE (total, adjusted prorated) |  | -.04 | .01 | .08* | .12** | -.06 | – |  |  |
| 7. CVD risk score |  | .06** | .02 | .08* | -.02 | -.00 | .03 | – |  |
| 8. Chronic conditions score |  | .25** | .09** | .21** | -.02 | .01 | .07 | .16** | – |
|  |  |  |  |  |  |  |  |  |  |

*Note. Sample size differs for each individual correlation, depending on the two variables in the analysis.*

**p* < .05, two–tailed. ***p* < .01, two–tailed.

**SUPPLEMENTARY TABLE D.** **Logistic regression models predicting lifetime abstinence from alcohol in the Sydney Memory and Ageing Study cohort**

|  | **A. Self-report apathy (n = 897)** | | | **B. Informant-report apathy (n = 857)** | | | |  |
| --- | --- | --- | --- | --- | --- | --- | --- | --- |
|  | ***ΔX2*** | ***OR*** | ***95% CI*** | | ***ΔX2*** | ***OR*** | ***95% CI*** | |
| **Model 1 Apathy** | 2.33 |  |  | | 1.98 |  |  | |
| Self–Report (GDS-3A) |  | 1.27 | .93, 1.73 | |  | N/A | N/A | |
| Informant-Report (NPI) |  | N/A | N/A | |  | .00 | .00, N/A | |
|  |  |  |  | |  |  |  | |
| **Model 2 Depression & fatigue** | 1.36 |  |  | | 3.64 |  |  | |
| Self-Report Depression (GDS-12D) |  | .97 | .80, 1.18 | |  | 1.03 | .85, 1.26 | |
| Self-Report Fatigue (AQoL-6D) |  | 1.28 | .85, 1.93 | |  | 1.39 | .95, 2.03 | |
|  |  |  |  | |  |  |  | |
| **Model 3 Interactions** | 0.33 |  |  | | 8.45 |  |  | |
| Apathy x depression |  | .98 | .81, 1.19 | |  | 0.01 | .00, N/A | |
| Apathy x fatigue |  | 1.02 | .66, 1.56 | |  | .82 | .00, N/A | |
| Apathy x sex |  | 1.22 | .59, 2.51 | |  | 5.00 | .00, N/A | |
|  |  |  |  | |  |  |  | |
| **Model 4 Health status** | 2.86 |  |  | | 3.04 |  |  | |
| CVD risk index |  | .95 | .87, 1.03 | |  | .94 | .86, 1.02 | |
| Chronic condition index |  | .99 | .96, 1.02 | |  | .99 | .96, 1.03 | |

*Note.* Dependent variable reference group are those who reported not abstaining from alcohol across the lifetime. Model 1 included sex, age, non-English speaking background, education, marital status, living situation, occupation, global cognition, *APOE* Ɛ4 allele carriage and vision impairment. Model 1 also included apathy (self-report part A, informant-report part B). Model 2 included depression and fatigue. Model 3 included interactions: apathy x depression, apathy x fatigue and apathy x sex. Model 4 included CVD risk index and chronic condition index.

Abbreviations: AQoL-6D = Assessment of Quality of Life-6D. GDS = Geriatric Depression Scale. NPI = NeuroPsychiatric Inventory.

****p* < .001, two–tailed. ***p* < .01, two–tailed. **p* < .05, two–tailed.

**SUPPLEMENTARY TABLE E. Logistic regression models predicting past period of heavier alcohol consumption in the Sydney Memory and Ageing Study cohort**

|  | **A. Self-report apathy (n = 892)** | | | **B. Informant-report apathy (n = 852)** | | | |  |
| --- | --- | --- | --- | --- | --- | --- | --- | --- |
|  | ***ΔX2*** | ***OR*** | ***95% CI*** | | ***ΔX2*** | ***OR*** | ***95% CI*** | |
| **Model 1 Apathy** | 1.35 |  |  | | 0.35 |  |  | |
| Self–Report (GDS-3A) |  | 1.09 | .94, 1.27 | |  | N/A | N/A | |
| Informant-Report (NPI) |  | N/A | N/A | |  | .93 | .71, 1.21 | |
|  |  |  |  | |  |  |  | |
| **Model 2 Depression & fatigue** | 2.32 |  |  | | 1.81 |  |  | |
| Self-Report Depression (GDS-12D) |  | 1.08 | .97, 1.19 | |  | 1.05 | .95, 1.17 | |
| Self-Report Fatigue (AQoL-6D) |  | 1.01 | .82, 1.25 | |  | 1.06 | .87, 1.30 | |
|  |  |  |  | |  |  |  | |
| **Model 3 Interactions** | 6.25 |  |  | | 16.65 |  |  | |
| Apathy x depression |  | 1.05 | .94, 1.15 | |  | 8.75 | 1.15, 66.82 | |
| Apathy x fatigue |  | .84 | .68, 1.04 | |  | .48 | .14, 1.60 | |
| Apathy x sex |  | 1.33 | .99, 1.79 | |  | 1.01 | .20, 5.19 | |
|  |  |  |  | |  |  |  | |
| **Model 4 Health status** | 3.42 |  |  | | 3.42 |  |  | |
| CVD risk index |  | 1.03 | .98, 1.07 | |  | 1.03 | .98, 1.08 | |
| Chronic condition index |  | 1.01 | .99, 1.03 | |  | 1.01 | .99, 1.03 | |

*Note.* Dependent variable reference group are those who reported not having had a past period of heavier alcohol consumption. Model 1 included sex, age, non-English speaking background, education, marital status, living situation, occupation, global cognition, *APOE* Ɛ4 allele carriage and vision impairment. Model 1 also included apathy (self-report part A, informant-report part B). Model 2 included depression and fatigue. Model 3 included interactions: apathy x depression, apathy x fatigue and apathy x sex. Model 4 included CVD risk index and chronic condition index.

Abbreviations: AQoL-6D = Assessment of Quality of Life-6D. GDS = Geriatric Depression Scale. NPI = NeuroPsychiatric Inventory.

****p* < .001, two–tailed. ***p* < .01, two–tailed. **p* < .05, two–tailed.

**SUPPLEMENTARY TABLE F. Logistic regression models predicting current smoking in the Sydney Memory and Ageing Study cohort**

|  | **A. Self-report apathy (n = 897)** | | | **B. Informant-report apathy (n = 857)** | | | |  |
| --- | --- | --- | --- | --- | --- | --- | --- | --- |
|  | ***ΔX2*** | ***OR*** | ***95% CI*** | | ***ΔX2*** | ***OR*** | ***95% CI*** | |
| **Model 1 Apathy** | 0.10 |  |  | | 0.11 |  |  | |
| Self–Report (GDS-3A) |  | 1.00 | .73, 1.55 | |  | N/A | N/A | |
| Informant-Report (NPI) |  | N/A | N/A | |  | .85 | .27, 2.68 | |
|  |  |  |  | |  |  |  | |
| **Model 2 Depression & fatigue** | 10.28 |  |  | | 7.11 |  |  | |
| Self-Report Depression (GDS-12D) |  | 1.16 | .99, 1.36 | |  | 1.15 | .95, 1.38 | |
| Self-Report Fatigue (AQoL-6D) |  | 1.69* | 1.03, 2.78 | |  | 1.54 | .96, 2.46 | |
|  |  |  |  | |  |  |  | |
| **Model 3 Interactions** | 1.31 |  |  | | 1.31 |  |  | |
| Apathy x depression |  | 1.02 | .86, 1.21 | |  | .00 | .00, N/A | |
| Apathy x fatigue |  | 1.17 | .71, 1.94 | |  | 1.15 | .04, 31.06 | |
| Apathy x sex |  | .77 | .37, 1.60 | |  | N/A | .00, N/A | |
|  |  |  |  | |  |  |  | |
| **Model 4 Health status** | 40.90 |  |  | | 38.30 |  |  | |
| CVD risk index |  | 1.51 | 1.31, 1.75 | |  | 1.50 | 1.30, 1.71 | |
| Chronic condition index |  | .98 | .93, 1.03 | |  | .98 | .93, 1.03 | |

*Note.* Dependent variable reference group are current non-smokers. Model 1 included sex, age, non-English speaking background, education, marital status, living situation, occupation, global cognition, *APOE* Ɛ4 allele carriage and vision impairment. Model 1 also included apathy (self-report part A, informant-report part B). Model 2 included depression and fatigue. Model 3 included interactions: apathy x depression, apathy x fatigue and apathy x sex. Model 4 included CVD risk index and chronic condition index.

Abbreviations: AQoL-6D = Assessment of Quality of Life-6D. GDS = Geriatric Depression Scale. NPI = NeuroPsychiatric Inventory.

****p* < .001, two–tailed. ***p* < .01, two–tailed. **p* < .05, two–tailed.

**SUPPLEMENTARY TABLE G. Logistic regression models predicting lifetime history of smoking in the Sydney Memory and Ageing Study cohort**

|  | **A. Self-report apathy (n = 896)** | | | **B. Informant-report apathy (n = 857)** | | | |  |
| --- | --- | --- | --- | --- | --- | --- | --- | --- |
|  | ***ΔX2*** | ***OR*** | ***95% CI*** | | ***ΔX2*** | ***OR*** | ***95% CI*** | |
| **Model 1 Apathy** | 1.86 |  |  | | 0.37 |  |  | |
| Self–Report (GDS-3A) |  | 1.11 | .96, 1.29 | |  | N/A | N/A | |
| Informant-Report (NPI) |  | N/A | N/A | |  | 1.10 | .79, 1.55 | |
|  |  |  |  | |  |  |  | |
| **Model 2 Depression & fatigue** | 1.24 |  |  | | 1.32 |  |  | |
| Self-Report Depression (GDS-12D) |  | 1.02 | .93, 1.13 | |  | 1.02 | .92, 1.13 | |
| Self-Report Fatigue (AQoL-6D) |  | 1.10 | .89, 1.37 | |  | 1.10 | .90, 1.34 | |
|  |  |  |  | |  |  |  | |
| **Model 3 Interactions** | 1.60 |  |  | | 5.66 |  |  | |
| Apathy x depression |  | .96 | .86, 1.06 | |  | .95 | .79, 1.15 | |
| Apathy x fatigue |  | 1.04 | .84, 1.29 | |  | 2.22 | .99, 4.95 | |
| Apathy x sex |  | 1.15 | .85, 1.56 | |  | .72 | .26, 1.97 | |
|  |  |  |  | |  |  |  | |
| **Model 4 Health status** | 3.16 |  |  | | 4.58 |  |  | |
| CVD risk index |  | 1.04 | 1.00, 1.09 | |  | 1.05 | 1.00, 1.10* | |
| Chronic condition index |  | 1.00 | .98, 1.02 | |  | 1.00 | .99, 1.02 | |

*Note.* Dependent variable reference group are those who reported not regularly having smoked in their lifetime. Model 1 included sex, age, non-English speaking background, education, marital status, living situation, occupation, global cognition, *APOE* Ɛ4 allele carriage and vision impairment. Model 1 also included apathy (self-report part A, informant-report part B). Model 2 included depression and fatigue. Model 3 included interactions: apathy x depression, apathy x fatigue and apathy x sex. Model 4 included CVD risk index and chronic condition index.

Abbreviations: AQoL-6D = Assessment of Quality of Life-6D. GDS = Geriatric Depression Scale. NPI = NeuroPsychiatric Inventory.

****p* < .001, two–tailed. ***p* < .01, two–tailed. **p* < .05, two–tailed.
